# Higher prevalence of dental caries and periodontal problems among refugees: A scoping review

**DOI:** 10.7189/jogh.13.04111

**Published:** 2023-09-15

**Authors:** Seyed Ahmad Banihashem Rad, Marcella Esteves Oliveira, Anastasia Maklennan, Paolo Castiglia, Guglielmo Campus

**Affiliations:** 1 Department of Restorative, Preventive and Pediatric Dentistry, University of Bern, Bern, Switzerland; 2 Graduate School for Health Sciences, University of Bern, Bern, Switzerland; 3 Department of Restorative Dentistry and Endodontology, Justus-Liebig-University Giessen, Giessen, Germany; 4 Department of Medicine, Surgery and Pharmacy, University of Sassari, Italy; 5 School of Dentistry, University of Sassari, Italy; * Joint first authorship.

## Abstract

**Background:**

We assessed the prevalence data on oral health diseases, namely dental caries and periodontitis, among refugees and asylum seekers worldwide.

**Methods:**

A systematic search of Scopus, Embase, and PubMed retrieved 1225 records; following title and abstract screening, 58 studies remained for full-text eligibility screening based on pre-defined inclusion criteria. Twenty-six studies were included in the review.

**Results:**

Dental caries and tooth loss due to caries were high in refugee populations, regardless of their age, gender, or nationality. The adult population had a mean decayed, missing, and filled teeth (DMFT) index score of 9.2 (standard deviation (SD) = 2.3); children had a score of 3.1 (SD = 1.1) for deciduous teeth and 2.5 (SD = 1.1) for permanents. Caries prevalence among refugees ranged from 4.6% to 98.7%, and gingivitis from 5.7% to 100%, indicating a high heterogeneity in their oral health. Regarding oral health accessibility, 17% to 72% of refugees had never been to a dentist, showing a very low level of accessibility to dental health services.

**Conclusions:**

Interventions and policies need to be designed to reduce oral health inequalities among refugee populations and asylum seekers, and host countries must implement strategies to increase their access to oral health care. Existing data should be used to set priorities for improving the oral health of refugees.

**Registration:**

Open Science Framework: https://doi.org/10.17605/OSF.IO/SU59K.

According to the International Organization for Migration (IOM), a refugee is an individual outside the country of his/her nationality and unable to benefit from its protection [1]. All refugees were once asylum seekers; although used interchangeably, these terms are not synonymous. Asylum-seekers seek international protection but have not yet been legally recognized as refugees.

According to the United Nations High Commissioner for Refugees (UNHCR), the number of displaced people worldwide has risen from 70.8 million in 2018 to a 100 million by 2022 [2,3], likely due to the situation in Afghanistan following withdrawal of the USA and new conflicts in Ukraine, Ethiopia, and Myanmar [4].

Refugee populations are at increased risk of developing a range of physical, psychological, and social health problems due to traumatic experiences and settlement pressures [5,6], as well as specific health problems [7], as many refugees come from countries where health systems have been damaged by conflict or civil unrest. Consequently, the prevalence of oral health problems is expected to be high among refugees [8], yet there is limited data and research to inform policymakers about their oral health needs and those of asylum seekers [9,10].

Oral diseases (i.e. dental caries and periodontitis) are a major contributor to the global burden of chronic disease [11,12]. Poor oral health negatively impacts quality of life and can increase the risk of developing chronic diseases [13]. For example, prolonged discomfort from an infected tooth can affect food intake and therefore nutrition. Additionally, bacteria associated with chronic periodontitis have been linked to diabetes and cardiovascular disease [14,15].

Refugee children are more likely to have poor oral health, impacting their overall health and well-being [16,17] and possibly causing malnutrition due to dietary changes and phonation difficulties, not only in children [18], but also in the elderly population [19]. Additionally, poor oral health might lead to an increase in body dissatisfaction [20] and negatively influence simple actions such as smiling, speaking, and eating [21]. Thus, oral health influences both overall health and mental health.

In summary, oral health diseases are among the most neglected aspects of health, regardless of location, culture, education, or economic standing, particularly in low- and middle-income countries. Thus, gaining a holistic overview of the prevalence of oral health problems among refugees and asylum seekers might assist policymakers in defining treatment needs and treatment strategies, as well as the best ways to adapt them to the host countries’ health systems, which are frequently overloaded when many refugees suddenly entering the country.

We aimed to conduct the first review on dental caries and periodontal problems in the refugee population on a global scale. Our main goals were to synthesize the evidence of the prevalence of dental caries among refugees and asylum seekers by evaluating the Decayed, Missing, and Filled Teeth in permanent and primary dentition (DMFT/dmft) index and to evaluate the prevalence of periodontal problems. We also aimed to appraise the dental care services provided to refugees and their needs and deficiencies.

## METHODS

We registered the scoping review at the Open Science Framework (OSF) registries (https://doi.org/10.17605/OSF.IO/SU59K). We conducted and reported the review following the Preferred Reporting Items for Systematic Reviews and Meta-Analyses (PRISMA) 2020 statement [22].

### Search strategy and selection criteria

#### Research question

Our research question was “What is the prevalence of dental caries and periodontal diseases among refugees and asylum seekers worldwide, and do they have an increased prevalence of these diseases than the general population of the host country?”, outlined based on the sample, phenomenon of interest, design, evaluation, and research type (SPIDER) [23] tool. We searched Scopus, Embase, and PubMed using a pre-designed search strategy; a representative search string for PubMed is presented here, while the remaining ones can be found in the **Online Supplementary Document**).

Sample (S): ((“Emigrants AND Immigrants”[MeSH Terms] OR “Undocumented Immigrants”[MeSH Terms] OR (“Refugees”[MeSH Terms] OR “Refugee Camps”[MeSH Terms]) OR “Ethnicity”[MeSH Terms] OR “Ethnic and Racial Minorities”[MeSH Terms] OR “asylum seeker*”[Title/Abstract] OR “displaced person*”[Title/Abstract] OR “refugee*”[Title/Abstract])

Phenomenon (P) of Interest (I): All the articles that related to either dental caries or periodontal problems.

Design (D): not restricted.

Evaluation (E): ((“Dental Caries”[MeSH Terms] OR “Root Caries”[MeSH Terms] OR “Dental Caries Susceptibility”[MeSH Terms] OR “Periodontal Pocket”[MeSH Terms] OR “Periodontal Index”[MeSH Terms] OR “Gingivitis”[MeSH Terms] OR “DMF Index”[MeSH Terms] OR “dmf index*”[Title/Abstract] OR “dental decay*”[Title/Abstract] OR “carious lesion*”[Title/Abstract] OR “Carious white spot*”[Title/Abstract] OR “periodontal pocket*”[Title/Abstract] OR “dmft s*”[Title/Abstract] OR “gingival index*”[Title/Abstract] OR “dmft*”[Title/Abstract] OR “dmft index*”[Title/Abstract] OR “bleeding on probing*”[Title/Abstract] OR “probing pocket depth*”[Title/Abstract] OR “clinical attachment loss*”[Title/Abstract])

Research type (R): not restricted.

#### Eligibility criteria

We included all quantitative and qualitative studies on dental caries or periodontal problems of refugees and asylum seeker populations published from 2011 to December 2022. We excluded non-peer-reviewed papers and unpublished research (e.g. theses, abstracts, and preprints).

#### Search strategy

**Figure 1:**
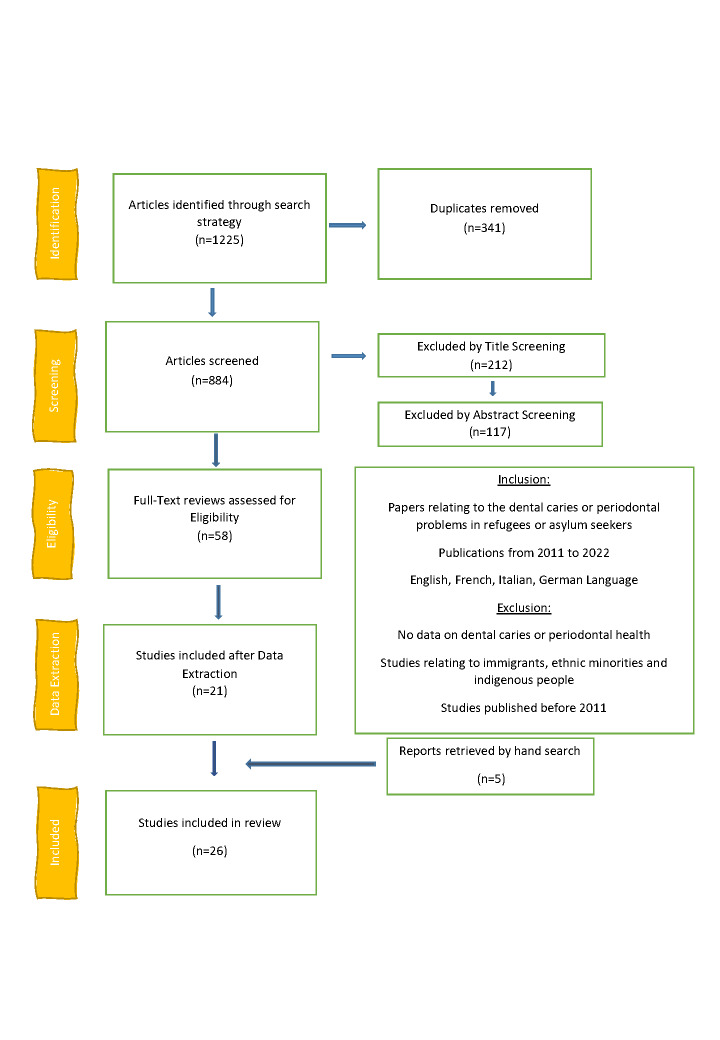
PRISMA flow diagram of papers selection.

Authors extracted text words and index keywords from relevant papers' titles and abstracts to identify relevant articles, developing search strings the selected keywords and synonyms in conjunction with the Boolean operators “AND” and “OR”. We only considered papers published in English, Italian, German, Spanish, and French. All age groups were included. The inclusion and exclusion criteria are displayed in **Figure 1**.

#### Study selection

Following deduplication, two independent reviewers (SABR and AM) screened the retrieved titles and abstracts (n = 884) to determine their relevance against pre-defined eligibility criteria, after which they examined the full texts of the selected studies (n = 58) for inclusion. If discrepancies between the two reviewers could not be resolved through discussion, a third reviewer (GC) was consulted.

#### Risk of bias

After excluding ineligible papers, two reviewers (SABR and AM) independently critically rated all eligible full texts using a critical appraisal instruments for prevalence studies in the Joanna Briggs Institute (JBI) System for the Unified Management of the Assessment and Review of Information (SUMARI) software (Joanna Briggs Institute, Adelaide, Australia) (**Online Supplementary Document**). Disagreements were resolved through discussion or the assistance of a third reviewer (GC). The appraisal instrument comprised nine questions to which the answers were “yes”, “no”, and “unclear.”

#### Data extraction and data synthesis

One author (SABR) extracted the following information using a pre-designed data collection form in Excel (Microsoft Corporation, Washington, USA): study characteristics – first author's last name, year of publication, country of study, study design, sampling procedures, calculation of sample size, and methods of data collection, participant characteristics and outcome measure – number of participants, sex, age, prevalence of dental caries and periodontal problems, oral health accessibility, and other findings from the original papers. The data was checked by a co-author (GC).

#### Parameters measured in the review

We excluded immigrants and ethnic minorities, as we focused on summarising data on oral health diseases (ie, dental caries and periodontitis) among refugee and asylum seeker populations.

In line with the World Health Organization (WHO) methodology [24,25], we applied the DMFT index score to evaluate dental caries [25], calculating the mean and standard deviation (SD) of the prevalence and range of dental caries scores where relevant. We considered both studies with prevalence (% of DMFT >0) and severity (mean DMFT) data on either permanent or deciduous dentition or periodontal problems (e.g. gingivitis, periodontitis).

## RESULTS

### Study selection

We retrieved 850 studies from Scopus, 98 from Embase, and 277 from PubMed. Following deduplication (n = 341), two reviewers (SABR, AM) screened the study titles, followed by their abstract (n = 212), after which 58 studies remained for full-text review (**Figure 1** and **Online Supplementary Document**). We then extracted data from 21 articles on refugees’ and asylum seekers’ oral health. We further retrieved five studies [26-30] through additional manual searches. Finally, we included 26 studies in the analysis [26-52] (**Table 1** and **Online Supplementary Document**).

**Table1:** List of studies included in review, ordered alphabetically by country where the study was conducted

	Year of study	Study type	Country of study	Country of origin of study participants	Number of participants	Age range in years
Nicol et al. [31]	2012	Cross-sectional	Australia	Afghanistan, Burma, Iran, Iraq, and Sri Lanka	105	0.7-6.7
Marwaha et al. [32]	2017	Cross-sectional	Australia	Afghanistan, Burma, Iran, Pakistan, and Sri Lanka.	201	18-74
van Berlaer et al. [26]	2015	Cross-sectional:	Belgium	Afghanistan, Iraq, Morocco	3907	0-75+
Hoover et al. [33].	2012	Pilot Study	Canada	The Indian subcontinent, other parts of Asia, and the rest of the world	89	3-15
Moreau et al. [34]	2013	Retrospective study	Canada	Africa, Europe, Middle East, North America, and South America,	120	1-14
Azrak et al. [35]	2017	Cross-sectional	Canada	Africa, Eastern Mediterranean, and South East Asia	211	1-5.9
Goetz et al. [36]	2016	Cross-sectional:	Germany	Afghanistan, Armenia, Chechnya, Eritrea, Iran, Iraq, Somalia, Syria, and Yemen	102	16-64
Solyman et al [37]	2016	Cross-sectional	Germany	Iraq and Syria	386	18-60
Takriti et al. [38]	2016	Cross-sectional	Germany	Afghanistan, Iraq, and Syria	288	18-75
Al-Ani et al. [39]	2016	Cross-sectional	Germany	Mainly from Syria, Afghanistan, Iraq, Eastern Europe, and from Asia (Iran, Pakistan, Thailand, Azerbaijan, Tajikistan, Russia,) as well as from African countries (Eritrea, Ghana, Nigeria, Ethiopia, and Somalia)	544	3-75+
Freiberg et al. [27]	2019	Retrospective Study	Germany	Afghanistan, Iran, Somalia, and Syria	568	20-34
Pavlopoulou et al. [28]	2010	Cross-sectional:	Greece	Afghanistan, Bangladesh, DR Congo, Eritrea, Iran, Kenya, Lebanon, Pakistan, Somalia, and Sudan	300	0-14
Kakalou et al. [29]	2015	Cross-sectional:	Greece	Afghanistan, Iraq, and Syria; other regions: Africa, Asia, and the Middle East	6688	0-75+
Bhatt et al. [40]	2017	Cross-sectional	India	Tibet	254	6-18
Noaman et al. [41]	2017	Cross-sectional	Iraq	Syria	79	4-5
Hamid et al. [42]	2020	Cross-sectional	Iraq	Syria	200	25-65
Biscaglia et al. [43]	2016	Cross-sectional	Palestine	Palestine (UNRWA Schools) Jordan, Lebanon, Syria, Gaza Strip, and West Bank	1550	6-18
Makan et al. [44]	2017	Cross-sectional	Jordan	Syria	125	6-12
Salim et al. [45].	2019	Cross-sectional	Jordan	Syria	606	7-19
Salim et al. [46]	2019	Cross-sectional	Jordan	Syria	547	18-50^+^
Joury et al. [47]	2017	Cross-sectional	Lebanon	Syria	823	4-23
Høyvik et al. [48]	2013	Cross-sectional	Norway	Middle East and Africa (Syria, Iran, Iraq, Afghanistan, Eritrea, Somalia, Sudan, Nigeria)	132	18^+^
Riatto et al. [49]	2015	Cross-sectional	Spain	Syria	156	5-13
Hjern et al. [30]	2015	Cross-sectional	Sweden	Afghanistan and Syria	639	6-15
Kazwini et al. [50]	2019	Cross-sectional	Syria	Syria	118	4-60
Flynn et al. [51]	2020	Cross-sectional	USA	Somalia	366	0.5-60

### Quality assessment

We did not exclude any study based on methodological quality assessment. The frame and adequacy of the sample size of the included studies received the lowest scores: five in two studies [44,50] and six in four studies [33,41,42,49] because there was no description of the sampling frame, participant selection procedures, and sample size calculation. Only 11 studies reported procedures for calculating sample size or its acceptability for the target group. Twenty studies provided a detailed description of the study setting and participants. Seven studies [38,41,42,44,48-50] failed to indicate the confidence interval (CI) for the mean value.

### Characteristics of included studies

Seven studies had a control group [28,30,33,34,38,39,50], primarily the host country’s local population, except for two studies [28,33] which had the immigrant population as a control group. Four included studies [33,38,44,48] assessed the treatment need of refugees, seven [33-35,38,40,41,46] reported on the utilisation of oral health services, two [41,43] investigated dietary factors, and two [41,51] investigated the mother-child caries rates, with one [51] showing a positive correlation between mother-child caries experience. Five studies [26,27,30,32,38] also included asylum seekers as participants. Three of these studies used the terms “refugee” and “asylum seeker” interchangeably [17,32,38]. None of the included studies had access to the oral health status of the sample group prior to their arrival.

**Figure 2:**
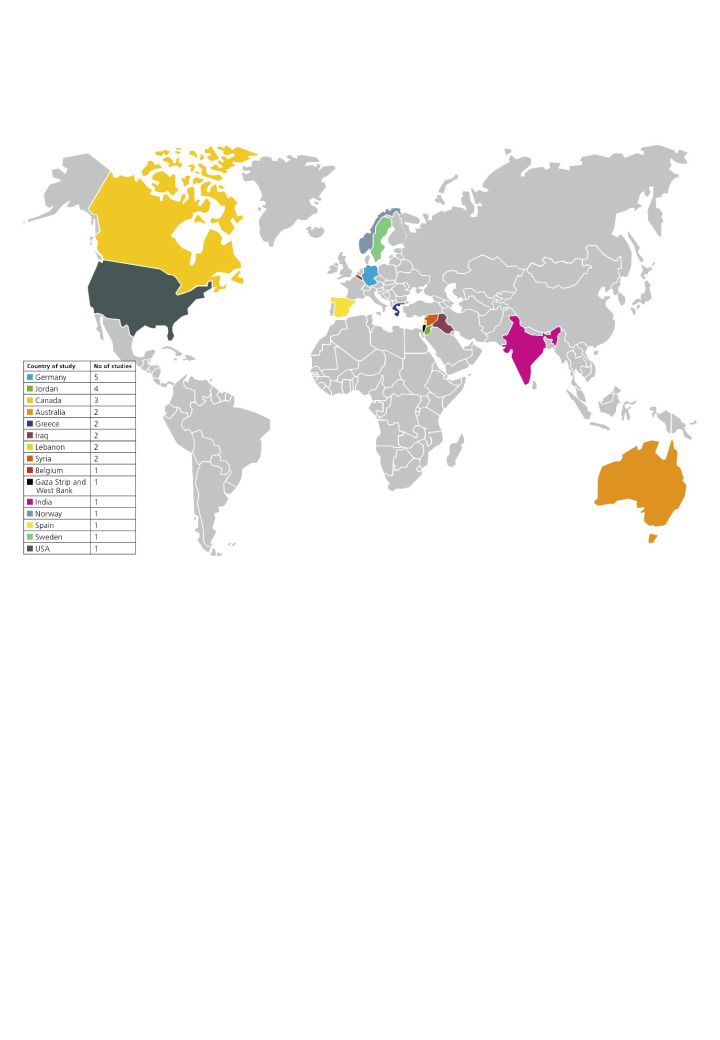
World map showing the countries, where studies regarding the oral health of refugees have been performed. A table at the left side shows additionally the number of studies per country, with the countries sorted by number of studies (from the highest to the lowest), where it is clearly observable the highest number of studies on refugees have been conducted in Germany. Some studies were conducted in more than one Country. Countries in which no studies could be found are marked in gray.

Children were the study populations in 11 studies [28,30,31,33-35,40,41,43,44,49], children and adults in eight [26,29,36,39,45,47,50,51], and only adults in seven [27,32,37,38,42,46,48]. All study samples consisted of more men than women, except for three studies [29,46,50]. Only three studies [38,39,43] had a multicenter clinical design. The main host countries were Germany, Canada, Australia, Jordan, and Iraq, while refugees originated from a wide range of countries, with most coming from Syria, Afghanistan, and Iraq (**Table 1**, **Figure 2**).

Age was directly correlated with caries prevalence [31,34,37]. In another study, the same trend was observed just for permanent dentition and an opposite relationship was shown for deciduous teeth. Meaning caries was inversely related to the age of the deciduous dentition [49] (**Online Supplementary Document**). Three studies [37,46,50] indicated that men had a lower oral health status (decayed and missing tooth) compared to women. This difference was significant in two studies [37,46] and not significant in one [50]. No substantial significant gender differences in dental status were found in five studies [31,41,48-50].

Country of origin was described a significant determinant for caries [33]. Refugees from the Middle East had more decayed teeth (DT) and a higher DMFT sum compared to refugees from Africa [48]. Refugees that had been displaced for more than five years were significantly more susceptible to caries than those who had been displaced for less than five years [47].

### Dental caries in refugees

Regarding caries in adults, four of the seven studies [38,39,42,46] indicated a very high DMFT severity (mean DMFT>10), while also showing an expanded version of the DMFT index with individual components (**Table 2**).

**Table 2:** Caries distribution in refugees in included studies*

	Sample size	DMFT	DT	MT	FT	dmft	dt	mt	ft	DMFT/dmft	DT/dt
**Adults**											
Solyman et al [37]	386	6.3 (5.0)	4 (3.3)	1.4 (3.3)	0.9 (1.6)	NA	NA	NA	NA	NA	NA
Goetz et al. [36]	102	6.89 (5.5)	2.9 (2.0)	3.8 (2.9)	3.7 (2.9)	NA	NA	NA	NA	NA	NA
Høyvik et al. [48]	132	7.4 (5.8)	4.3 (3.5)	1.4 (2.4)	1.7 (3.4)	NA	NA	NA	NA	NA	NA
Hamid et al. [42]	200	10.7 (3.3)	NA	NA	NA	NA	NA	NA	NA	NA	NA
Takriti et al. [38]	288	10.9 (3.7)	3.4 (0.3)	4.1 (3.1)	3.3 (1.0)	NA	NA	NA	NA	NA	NA
Al-Ani et al. [39]	544	10.9 (3.7)	3.4 (0.3)	4.1 (3.1)	3.3 (1.0)	NA	NA	NA	NA	NA	NA
Salim et al. [46]	547	11.8 (1.7)	5.4 (0.3)	3.5 (1.2)	3.4 (0.6)	NA	NA	NA	NA	NA	NA
Overall	2199	9.2 (2.3)	3.9 (0.8)	3(1.2)	2.7 (1.1)	NA	NA	NA	NA	NA	NA
**Children**											
Nicol et al. [31]	105	NA	NA	NA	NA	5.2 (4.1)	NA	NA	NA	NA	NA
Hoover et al. [33]	89	NA	NA	NA	NA	NA	NA	NA	1.5 (2.3)	5.8 (4.2)	3.0 (3.4)
Moreau et al. [34]	120	NA	NA	NA	NA	NA	NA	NA	NA	7.2 (5.1)	6.7 (5.0)
Azrak et al. [35]	211	NA	NA	NA	NA	2.2 (3.8)	1.7 (3.0)	0.2 (0.8)	0.3 (1.6)	NA	NA
Al-Ani et al. [39]	544	1.4 (1.2)	0.8 (0.8)	0.0.1 (0.1)	0.4 (0.3)	3.0 (1.8)	2.4 (1.4)	0.2 (0.2)	0.3 (0.2)	NA	NA
Bhatt et al. [40]	254	2.8 (2.4)	NA	NA	NA	3.5 (4.1)	NA	NA	NA	NA	NA
Noaman et al. [41]	79	NA	NA	NA	NA	2.9 (0.8)	NA	NA	NA	4.4 (5.0)	3.2 (4.3)
Salim et al. [45]	606	4.3	NA	NA	NA	NA	NA	NA	NA	NA	NA
Makan et al. [44]	125	3.6 (9.8)	NA	NA	NA	2.9 (4.7)	NA	NA	NA	NA	NA
Biscaglia et al. [43]	1550	2.5 (2.5)	NA	NA	NA	NA	NA	NA	NA	NA	NA
Joury et al. [47]	823	NA	2.0 (2.3)	NA	NA	NA	3.2 (3.0)	NA	NA	NA	5.3
Riatto et al. [49]	156	0.8 (1.8)	0.7 (1.79	0 (0.1)	0.1 (0.4)	2.2 (2.9)	NA	NA	NA	NA	NA
Kazwini et al. [50]	118	2.4 (2.3)	NA	0.8 (1.3)	0.7 (1)	NA	NA	NA	NA	2.48	NA
Overall	4780	2.5 (1.1)	0.7 (0.07)	0.4 (0.4)	0.4 (0.3)	3.1 (1.1)	2.4 (0.7)	0.2	0.7 (0.6)	5.8 (1.4)	4.3 (2.0)

DMFT – decaying, missing and filled index score in permanent dentition, dmft – caries index in primary dentition, DT – decaying teeth in permanent dentition, MT – missing teeth in permanent dentition, FT – filled teeth in permanent dentition, dt – decaying teeth in primary dentition, mt – missing teeth in primary dentition, ft – filled teeth in primary dentition, NA – not applicable, SD – standard deviation

*Data presented as mean (SD) unless otherwise specified.

DT was high in all but one study [49] in which the authors suggest that the high to moderate socioeconomic status of the sample population is the reason for lower DT. The overall DMFT mean SD obtained in our systematic search was 9.2 (SD = 2.3) for the adult population. The DT accounted for the highest proportion in the DMFT index, while the least proportion belonged to filled teeth (FT).

Regarding caries in children, two studies [31,34] reported very high decayed, missing, and filled teeth in primary dentition (dmft) of >5, with an overall mean dmft of 3.1 (SD = 1.1) for deciduous teeth and a DMFT of 2.5 (SD = 1.1) for permanent teeth. DT and dt in children/primary dentition (dt) also accounted for the highest proportion of caries in children's primary and permanent dentition. Overall, the mean DT among children, regardless of dentition stage, was 4.3 (SD = 2.0), which is relatively high (**Table 2**).

Only one study reported a higher prevalence of caries in local population compared to refugees [39], reporting that the German resident population has a slightly higher caries experience (11.2 vs 10.9), but a significantly lower treatment need compared to refugees.

### Caries prevalence and further detail of included papers

Most studies focused on oral health, except for four [26,28-30] which involved also general health. The prevalence of dental caries was higher in studies that focused on oral health. Only one study [49] revealed a low prevalence of caries, which was attributed to the fact that children were from wealthy households. The caries prevalence in primary dentition ranged from 55.8% to 84.0%, and in permanent dentition from 27.6% to 94.1%. Overall, the caries prevalence regardless of dentition stage ranged from 4.6% to 98.7% among the refugee population (**Table 3**).

**Table 3:** Further detail of included studies and caries prevalence

	Focus GH or OH	Dentist involved	Instruments mentioned	Reliability tested†	Caries detection method	Caries prevalence in %
**Primary dentition**						
Riatto et al. [49]	OH	Yes	Yes	Yes	WHO	55.8
Nicol et al. [31]	OH	Yes	NA	NA	NA	62
Noaman et al. [41]	OH	NA	Yes	Yes	WHO	64.5
Bhatt et al. [40]	OH	NA	Yes	Yes	WHO	84
**Permanent dentition**						
Riatto et al. [49]	OH	Yes	Yes	Yes	WHO	27.6
Takriti et al. [38]	OH	Yes	Yes	NA	WHO	53
Biscaglia et al. [43]	OH	NA	Yes	Yes	WHO	72.8
Bhatt et al. [40]	OH	NA	Yes	Yes	WHO	79.5
Marwaha et al. [32]	OH	Yes	Yes	Yes	ICDAS II	82
Solyman et al. [37]	OH	Yes	Yes	1Dentist	WHO	87.5
Høyvik et al. [48]	OH	Yes	Yes	Yes	astdd	89.4
Salim et al. [45]	OH	Yes	Yes	Yes	WHO	92.4
Salim et al. [46]	OH	NA	Yes	Yes	WHO	94.1
**Unspecified dentition**						
Kakalou et al. [29]	GH	NA	NA	NA	ICD-10	4.6
van Berlaer et al. [26]	GH	NA	NA	NA	ICD-10	8.1
Pavlopoulou et al. [28]	GH	NA	NA	NA	NA	24.7
Azrak et al. [35]	OH	Yes	Yes	NA	WHO	48.8
Hjern et al. [30]	GH	NA	Yes	NA	NA	48.1
Hoover et al. [33]	OH	Yes	Yes	NA	NA	67.4
Al-Ani et al. [39]	OH	Yes	Yes	Yes	WHO	79.5
Joury et al. [47]	OH	Yes	NA	NA	NA	90.2
Freiberg et al. [27]	OH	Yes	NA	NA	NA (BEMA)*	98.7
Moreau et al. [34]	OH	NA	Yes	NA	WHO	NA
Goetz et al. [36]	OH	Yes	Yes	1 dentist	ICDAS (STROBE)	NA
Hamid et al. [42]	OH	NA	Yes	NA	NR	NA
Makan et al. [44]	OH	Yes	NA	NA	WHO	NA
Kazwini et al. [50]	OH	Yes	Yes	NA	WHO	NA
Flynn et al. [51]	OH	NA	NA	NA	WHO	NA

GH – general health, OH – oral health, NA – not applicable, WHO – World Health Organization, ICD-10 – International Classification of Diseases 10^th ^revision, astdd – Association of State and Territorial Dental Directors, ICDAS – International Caries Detection and Assessment System, BEMA – Einheitliche Bewertungsmaßstab für zahnärztliche Leistungen [Scale of Evaluation for Medical Services in the Statutory Health Insurance Sector]

*BEMA is the standard of evaluation of dental services and forms within the statutory health insurance in Germany (dentist recorded caries in BEMA forms).

†Reliability tested: If the studies gave information about inter or intra reliability of dental examination, it is showed as Yes or NA. The studies that did not report the caries prevalence, reported caries in other forms DMFT/S.

### Other indices to report caries: Decayed-Missing-Filled Surfaces and International Caries Detection and Assessment System

Four studies [32,35,43,51] reported caries prevalence in other forms using Decayed-Missing-Filled Surfaces (DMFS) or International Caries Detection and Assessment System (ICDAS). Two studies [35,43] reported caries using DMFS, while one used ICDAS [53] (**Table 4**). Analysis of tooth surfaces found that white spot lesions were especially frequent in age groups 18-29 (mean = 4.45 (SD = 4.95)) and 30-39 (mean = 3.49 (SD = 4.74)) [32].

**Table 4:** Caries distribution in refugees*

	Age in years	DMFS	Decayed surfaces		Missing surfaces		Filled surfaces	
Azrak et al. [35]	1-5.9	4.8 (11.0)	3.0 (6.7)		0.7 (3.5)		1.1 (6.2)	
Biscaglia et al. [43]	6-18	3.99 (4.59)	3.29 (3.99)		0.22 (1.12)		0.48 (1.34)	
Flynn et al. [51]	<2	0 (0)	0 (0)		NA		0 (0)	
	2-5	2.3 (6.1)	0.8 (1.5)		NA		1.6 (6.0)	
	6-11	4.2 (8.2)	1.0 (2.9)		NA		3.3 (7.9)	
	12	0.8 (1.2)	0 (0)		NA		0.8 (1.2)	
	<35	20.1 (19.6)	5.2 (7.2)		9.8 (15.5)		5.1 (6.9)	
	>35	22.8 (18.3)	5.9 (9.3)		9.7 (13.0)		7.2 (9.1)	
**ICDAS study**	**Age in years**	**ICDAS**		**ICDAS codes 1-2**		**ICDAS codes 3-4**		**ICDAS codes 5-6**
Marwaha et al. [32]	18-29	7.40 (6.59)	4.45 (4.95)		1.80 (2.94)		1.27 (2.59)	
	30-39	6.27 (6.55)	3.49 (4.74)		1.73 (2.16)		0.87 (2.08)	
	40-49	5.78 (6.25)	2.88 (4.58)		1.47 (2.06)		0.92 (3.42)	
	≥50	4.47 (5.19)	2.24 (4.01)		0.76 (1.22)		1.47 (3.58)	

NA – not applicable, DMFS – decaying, missing, and filled surfaces, ICDAS – International Caries Detection and Assessment System

*Caries value is reported as mean (SD) unless otherwise specified.

### Periodontal health in refugees

Seven studies [27,31,33,34,42,44,49] examined periodontal health; all focussed on children, except two [27,42] which addressed periodontal health in adults. Regarding periodontal health in children, the prevalence of gingivitis ranged from 5.7% to 100%, indicating high asymmetry. The prevalence of gingivitis was reported as very high in three studies [33,34,44], with one study reporting that almost all children had chronic gingivitis [44] and two reporting a prevalence of two thirds [33,34]. Although gingival inflammation was apparently high [33,34,44], two studies reported a prevalence of gingivitis of 5.7% [31] and 14% [49].

Regarding periodontal health in adults, the prevalence of periodontitis was present in 2.8% of the observed population [27], with most cases diagnosed with apical periodontitis and a prevalence of gingivitis at 0.9%. The mean Gingival Index [54] was 0.8 (SD = 0.7) and ranged from 0 to 2.7 in the Iraq refugees [42].

All studies except one [35], reported that the prevalence of gingivitis in refugees was higher compared to the local population.

### Oral health accessibility

Access to oral health care is an important determinant of oral health status. Unfamiliarity with the health care system can make obtaining oral care difficult. Moreover, in some refugee-receiving countries, dental treatment can be financially unfeasible.

Studies indicate that refugees in the transition phase mainly receive emergency treatment. Once refugee status is granted, refugees often have better access to dental care [26,28,37,52]. According to three studies, general referral systems appeared to be in place [26,28,36]. None of these studies went into specifics about the utility of referral systems.

When asking adult refugees about the history of their last dentist visit, the percentage of individuals who never visited a dentist in their life ranged from 17% to 33% (**Online Supplementary Document**), increasing to between 42% and 72% among children, indicating very low levels of accessibility to dental health services in the country of origin and the low socio-economic level of many refugees.

## DISCUSSION

The included studies indicated that the prevalence of oral health problems among refugee populations was relatively high compared with the general population of host countries. Even though the perceived need for treatment varied between studies, dental caries and periodontal disease were most commonly perceived as urgent problems for refugees.

The high prevalence of dental caries and periodontal disease in this population, as well as limited access to oral health care, low utilisation of preventive oral health services, and the high cost of dental care, were the most common explanations for refugees having their teeth extracted when they could have been preserved under “conventional” conditions. Oral screening is not usually available as standard in host countries; consequently, detectable oral health problems remain undetected, increasing the likelihood of more invasive treatment at subsequent dental visits. An additional challenge is the lack of information on pre-arrival oral conditions, which makes difficult to compare and assess the progression of oral health conditions.

Oral health can be neglected due to pressing resettlement issues, as shown in almost all studies. Additionally, access to dental services and language barriers had been significant barriers to dental care for refugees [37,49]. Access to essential dental services can also be affected by language, cultural and economic barriers, social isolation, unfamiliarity with the local health system, laws, regulations and other constraints.

Refugees tend to be less motivated by and focused on oral health treatment and prevention than the native community [37] as they might prioritise resettlement [48].

Kakalou et al. [29] reported the lowest caries prevalence value among all included studies; however, this study focused on general rather than oral health, and the oral examinations were performed by medical clinicians and not dentists, which may have led to an underestimation of the prevalence of oral disorders.

Some studies [36,48] examined the effects of oral health on the general health of refugees. For example, Høyvik et al. [48] argued that dental disorders considerably impact social, physical, and mental health of this population. In this context, missing teeth have been reported to have a significant impact on refugees' self-confidence and ability to learn a new language. Moreover, dental anomalies in Western cultures can have a substantial effect on self-esteem, social conduct, employment, housing, and social impressions of others [55]. Reduced social and psychological well-being can delay the process of acceptance and integration, leading to social isolation and mental health problems that exacerbate general health problems [36].

Although the refugees have poor oral status, there is variation among subgroups, and children of African ancestry reported better oral health conditions than other refugee groups [16,48]. The proportion of caries-free children aged five to six years and 12 years was more than 80% [56]. The low levels of caries in the Ethiopian children may be due to most of the food consumed by Ethiopian refugees in their culture being produced without refined sugar. East African countries typically have low caries prevalence compared to industrialised countries, with rural areas having lower prevalence than urban areas. Furthermore, differences in caries prevalence between high and low socioeconomic categories have been reported, with caries prevalence and severity generally higher among wealthier Africans living in urban areas where sugar consumption is higher and considered a luxury [57].

Oral health problems are magnified when refugees begin their journey. Their pre-existing health conditions worsen during the journey and while waiting for official recognition of refugee status by the host country [37]. An important reason for the worsening of oral health is due to their diet changing in the host country [27,30]. Increased sugar consumption among refugees upon arrival in Europe has been observed [27], affecting their dental health. Children are particularly vulnerable, as their families traditionally promote caries-related dietary habits [30], Moreover, the lack of preventive measures adds up to the burden of oral problems to refugee children. One study reported that parents were unwilling to adopt preventive approaches to oral health [48] and only took their children to the dentist when they had pain.

Refugees' prior dental care experiences in their home countries and their beliefs could impact their dental hygiene practices in the host countries. Dental pain and fear of dental procedures reduced the likelihood of going to the dentist [39], which was exacerbated by linguistic barriers and the inability to express their emotions appropriately. This highlights the need of enhancing communication between physicians and refugees through the use of interpreters, when necessary, and the provision of informational pamphlets in the refugees' native language, particularly about diet and the effects of sugar on oral health [37].

Oral examination of refugees at the point of entry or registration for further dental screening could be a useful approach [48]. Communication in the native languages can also prevent miscommunication and delays [37]. Additionally, all refugee-hosting countries are called upon to enhance their dental care capacity, as the need for refugee dental services is likely to increase steadily in the near future. Despite all the developments and resources available in host countries, especially in European countries with developed health care system, the inclusion and integration of refugees and asylum seekers remains a challenge [36,48]. More studies are needed to understand the oral health perspectives of refugees and asylum seekers. Future research should focus on identifying specific characteristics and beliefs in order to develop targeted and efficient interventions to improve oral health status in displaced people.

One of the limitations of this study is that some information could have been missed, as the studies retrieved in the systematic database search showed huge differences in the characterization and reported data of the refugee population. We also observed significant differences in the sampling procedure, power calculation, and geographical location among the included studies; several [41,42,44,50] did not describe sample size calculation, and refugee populations were usually smaller than the general population. Moreover, refugee status dynamics and interactions could have affected the result.

We did not conduct a meta-analysis due to the lack of comparability among the studies and high heterogenicity. The limited number of publications on this topic, particularly from developing and underdeveloped countries, and the inclusion of studies published exclusively after 2011, reduces the generalisability of our findings. Human mistakes and bias, which may have led to losing some information or biasing the results, are also possible.

Despite these limitations, this study is, to the best of our knowledge, the first systematic global evaluation of dental caries and periodontal diseases, including from a quantitative perspective, in refugee populations. It adds to the limited existing knowledge on special needs and associations necessary for future planning to improve refugees’ oral health. Our findings have substantial implications for professionals working in the field of oral health as well as for oral public health efforts. Refugee populations constitute a small proportion of the population in the host countries, and inequalities in refugee oral health care are often masked in population-level data. The studies we examined here successfully addressed the reality of refugee oral health in their respective countries, and future studies should use identical comparative approaches to provide an accurate depiction of population oral health.

## CONCLUSIONS

There is more available data on refugees' general health needs and problems than regarding refugees oral health. The prevalence and severity of dental caries are higher among refugees and asylum seekers than among the local population in each host country, regardless of age, sex, or country. Rates of untreated dental caries (i.e. DT) and tooth loss (i.e. missing teeth) due to caries are higher in this population. A high prevalence of dental caries and limited access to dental care are major challenges faced by refugees and asylum seekers worldwide. Interventions and policies need to be designed to reduce oral health inequalities in this population, and host countries need to implement strategies to significantly increase access to oral health care for refugees and asylum seekers. Future studies need to add to real-world knowledge about refugee oral health, as they can help host-countries policy makers improve refugee oral health and develop a more cost-effective preventive approach to oral health care. There is an urgent need to use existing data to set priorities for improving the oral health of refugees.

## Additional material


Online Supplementary Document
