# Supplementary material for: Higher prevalence of dental caries and periodontal problems among refugees: A scoping review
Source: J Glob Health. 2023 Sep 15;13:04111. doi: 10.7189/jogh.13.04111 (PMC10503462; doi:10.7189/jogh.13.04111)
Supplement: Online Supplementary Document [file jogh-13-04111-s001.pdf]

Supplementary files for "Higher prevalence of Dental Caries and Periodontal problems among Refugees: A Scoping Review"

CONTENTS

Search strategy for each electronic database ....Error! Bookmark not defined.

Risk of bias assessment...Error! Bookmark not defined.

Excluded studies after the assessment of full texts and reasons for exclusion (N=37) 4

Relationship between caires prevalence and age in refugee children.. 5

History of dental visit 5

References of excluded studies 5

Appendix 1. Detailed search procedure in databases

Pubmed (n=277)	(("Emigrants AND Immigrants"[MeSH Terms] OR "Undocumented Immigrants"[MeSH Terms] OR ("Refugees"[MeSH Terms] OR "Refugee Camps"[MeSH Terms]) OR "Ethnicity"[MeSH Terms] OR "Ethnic and Racial Minorities"[MeSH Terms] OR "asylum seeker*"[Title/Abstract] OR "displaced person*"[Title/Abstract] OR "refugee*"[Title/Abstract]) AND (("Dental Caries"[MeSH Terms] OR "Root Caries"[MeSH Terms] OR "Dental Caries Susceptibility"[MeSH Terms] OR "Periodontal Pocket"[MeSH Terms] OR "Periodontal Index"[MeSH Terms] OR "Gingivitis"[MeSH Terms] OR "DMF Index"[MeSH Terms] OR "dmf index*"[Title/Abstract] OR "dental decay*"[Title/Abstract] OR "carious lesion*"[Title/Abstract] OR "Carious white spot*"[Title/Abstract] OR "periodontal pocket*"[Title/Abstract] OR "dmft s*"[Title/Abstract] OR "gingival index*"[Title/Abstract] OR "dmft*"[Title/Abstract] OR "dmft index*"[Title/Abstract] OR "bleeding on probing*"[Title/Abstract] OR "probing pocket depth*"[Title/Abstract] OR "clinical attachment loss*"[Title/Abstract]) AND ("english"[Language] OR "french"[Language] OR "german"[Language] OR "italian"[Language]))) AND ((english[Filter] OR french[Filter] OR german[Filter] OR italian[Filter]) AND (2011:2022[pdat]))
EMBASE (n=98)	Sources Embase, MEDLINE<br>Query('immigrant':ti,ab OR 'immigrants':ti,ab OR 'refugee':ti,ab OR 'refugee camp':ab OR 'asylum seeker':ti,ab OR 'ethnic group':ti,ab)<br>AND ('dental caries':ti,ab OR 'dmf index':ti,ab OR 'dmft index':ti,ab OR 'dmfs index':ti,ab OR 'caries assessment':ti,ab OR 'periodontal disease':ti,ab OR 'gingiva disease':ti,ab OR 'gingivitis':ti,ab OR 'periodontal pocket depth':ti,ab OR 'pocket depth':ti,ab OR 'gingival index':ti,ab OR 'bleeding on probing':ti,ab OR 'clinical attachment level':ti,ab OR 'decay rate':ti,ab) AND ([embase]/lim OR [medline]/lim OR [pubmed-not-medline]/lim) AND ([english]/lim OR [french]/lim OR [german]/lim OR [italian]/lim) AND [2011-2022]/
Scopus (n=850)	((immigrant* OR refugee* OR "refugee camp" OR "asylum seeker" OR "ethnic group")) AND (("dental caries" OR "DMF index" OR "DMFT index" OR "DMFS index" OR "caries" OR "periodontal disease" OR "gingiva disease" OR "gingivitis" OR "periodontal pocket depth" OR "pocket depth" OR "gingival index" OR "bleeding on probing" OR "clinical attachment level" OR "decay rate")) AND (LIMIT-TO (PUBYEAR , 2022) OR LIMIT-TO (PUBYEAR , 2021) OR LIMIT-TO (PUBYEAR , 2020) OR LIMIT-TO (PUBYEAR , 2019) OR LIMIT-TO (PUBYEAR , 2018) OR LIMIT-TO (PUBYEAR , 2017) OR LIMIT-TO (PUBYEAR , 2016) OR LIMIT-TO (PUBYEAR , 2015) OR LIMIT-TO (PUBYEAR , 2014) OR LIMIT-TO (PUBYEAR , 2013) OR LIMIT-TO (PUBYEAR , 2012) OR LIMIT-TO (PUBYEAR , 2011)) AND (LIMIT-TO (LANGUAGE , "English") OR LIMIT-TO (LANGUAGE , "German") OR LIMIT-TO (LANGUAGE , "French") OR LIMIT-TO (LANGUAGE , "Italian")) AND (LIMIT-TO (EXACTKEYWORD , "Oral Health") OR LIMIT-TO (EXACTKEYWORD , "Health"))

Appendix 2: Quality assessment (JBI SUMARI)

Citation	Q1	Q2	Q3	Q4	Q5	Q6	Q7	Q8	Q9	Total score
Nicol et al.(26)	Y	Y	N	Y	Y	Y	Y	Y	Y	8
Marwaha et al. (27)	U	Y	U	Y	Y	Y	Y	Y	Y	7
van Berlaer et al.(28)	U	Y	Y	Y	Y	U	Y	Y	Y	7
Hoover et al.(29)	U	Y	N	Y	Y	Y	Y	Y	N	6
Moreau et al.(30)	Y	Y	N	Y	Y	Y	Y	Y	Y	8
Azrak et al. (31)	Y	Y	N	Y	Y	Y	U	Y	Y	7
Goetz et al.(32)	Y	Y	N	Y	Y	Y	Y	Y	Y	8
Solyman et al.(33)	Y	Y	Y	Y	Y	Y	Y	U	Y	8
Takriti et al.(34)	Y	Y	U	Y	Y	Y	U	Y	Y	7
Al-Ani et al. (35)	Y	Y	Y	Y	Y	Y	Y	Y	Y	9
Freiberg et al.(36)	U	Y	Y	Y	Y	Y	Y	Y	Y	8
Pavlopoulou et al.(37)	U	Y	Y	Y	Y	Y	Y	Y	Y	8
Kakalou et al.(38)	U	Y	Y	Y	Y	Y	Y	Y	Y	8
Bhatt et al. (39)	U	Y	N	Y	Y	Y	Y	Y	Y	7
Noaman et al. (40)	Y	Y	N	Y	U	Y	Y	Y	N	6
Hamid et al.(41)	Y	U	U	Y	U	Y	Y	Y	Y	6
Biscaglia et al.(42)	Y	Y	Y	U	Y	Y	Y	Y	Y	8
Makan et al.(43)	U	Y	N	U	Y	Y	Y	Y	U	5
Salim et al. (44)	U	Y	Y	Y	Y	Y	Y	Y	Y	8
Salim et al.(45)	U	Y	Y	Y	Y	Y	Y	Y	Y	8
Joury et al.(46)	Y	Y	Y	U	Y	U	Y	Y	Y	7
Høyvik et al.(47)	Y	Y	U	Y	Y	Y	Y	U	Y	7
Riatto et al.(48)	N	Y	U	Y	Y	Y	Y	U	Y	6
Hjern and Kling (49)	N	Y	Y	Y	Y	Y	Y	Y	U	7
Kazwini et al.(50)	U	Y	N	Y	U	Y	Y	U	Y	5
Flynn et al.(51)	Y	Y	N	Y	Y	Y	Y	Y	Y	8
Total (%) yes	50	96.1	42.3	88.4	88.4	92.3	92.3	84.6	84.6	

Critical appraisal questions: Q1. Was the frame appropriate to address the target population? Q2. Were study participants sampled in an appropriate way? Q3. Was the sample size adequate? Q4. Were the study subjects and the setting described in detail? Q5. Was data analysis conducted with sufficient coverage of the identified sample? Q6. Were valid methods used for identification of the condition? Q7. Was the condition measured in a standard, reliable way for all participants? Q8. Was there appropriate statistical analysis? Q9. Was the response rate adequate and, if not, was the low response rate managed appropriately? Y, yes; U, unclear; N, no

Appendix 3: Excluded papers

Excluded Studies	REASON FOR EXCLUSION
Amin et al. (2015)	The study participants were immigrants.
Christian . (2015)	The study participants were immigrants.
Ferrazzano et al. (2019)	The study participants were immigrants.
Gibbs et al. (2016)	The study participants were immigrants.
Hamid et al. (2020)	The study participants were immigrants.
Hashizume et al. (2011)	The study participants were immigrants.
Lin et al.(2019)	The study participants were immigrants.
Lin et al.(2014)	The study participants were immigrants.
Liu C et al. (2016)	The study participants were immigrants.
Olerud et al (2016)	The study participants were immigrants.
Sivakumar et al.(2016)	The study participants were immigrants.
Stecksén-Blicks et al.(2014)	The study participants were immigrants.
Wilson et al. (2018)	The study participants were immigrants.
Dahlan et al. (2021).	The study participants were immigrants.
Diamanti et al. (2022)	The study participants were immigrants.
Gómez- et al. (2021)	The study participants were immigrants.
Kabani et al. (2020)	The study participants were immigrants.
Sanders et al. (2020)	The study participants were immigrants.
Thorbert-Mros et al. (2021)	The study participants were immigrants.
Traisuwan et al. (2021)	The study participants were immigrants.
Zhang et al. (2013)	The study participants were ethnic minorities.
Caplin et al. (2015)	The study participants were immigrants.
Chen et al. (2021)	The study participants were ethnic minorities.
Premaraj et al. (2020)	The study participants were ethnic minorities.
Sgan-Cohen et al. (2014)	The study participants were ethnic minorities.
Shi et al. (2018)	The study participants were ethnic minorities.
Van Der Tas et al. (2016)	The study participants were ethnic minorities.
Wu et al. (2021)	The study participants were ethnic minorities.
Wulaerhan et al. (2014)	The study participants were ethnic minorities.
Zhang et al. (2014)	The study participants were ethnic minorities.
Zhang et al (2015)	The study participants were ethnic minorities.
Hermans et al. (2017)	No information about DMFT or Caries prevalence.
Williams et al. (2016)	No information about DMFT or Caries prevalence.
Mattila et al. (2016)	No separate information about DMFT or Caries prevalence.
Vega-López et al.(2018)	No information about DMFT or Caries prevalence.
Ogawa et al. (2019)	No information on prevalence and only indicating the caries risk.
Alrashdi et al. (2021)	No information about DMFT or Caries prevalence.

Appendix 4: Oral health status of refugee children within the distinct age groups. .

According to the data obtained from Riatto et al.[48]

Variables	Age groups (years)		
	5–7	8–10	11–13
Global caries prevalence (DMFT or dft > 1) (% of subjects)	74.5	74.2	59.0
Caries prevalence in permanent dentition (% of subjects)	7.8	34.8	41.0
Caries prevalence in deciduous dentition (% of subjects)	72.5	60.6	25.6

History of dental visits

Author	Azrak et al.[31]	Moreau et al.[31]	Bhatt et al.[39]	Noaman et al. [40]	Salim et al.[44]	Høyvik et al.[47]
	Children				*Adults*	
Never visited a dentist (%)	*72.7*	*60*	*60*	*42*	*17*	*33.3*

Appendix 5: History of dental visit

Appendix 6: Reference list of excluded studies

1. Amin MS, Perez A, Nyachhyon P. Parental awareness and dental attendance of children among African immigrants. J Immigr Minor Health. 2015;17(1):132-8.
2. Christian B, Young D, Gibbs L, De Silva A, Gold L, Riggs E, et al. Exploring child dental service use among migrant families in metropolitan Melbourne, Australia. Australian Dental Journal. 2015;60(2):200-
3. Ferrazzano GF, Cantile T, Sangianantoni G, Ingenito A, Rengo S, Alcidi B, et al. Oral health status and Unmet Restorative Treatment Needs (UTN) in disadvantaged migrant and not migrant children in Italy. European Journal of Paediatric Dentistry. 2019;20(1):10-4.
4. Gibbs L, de Silva AM, Christian B, Gold L, Gussy M, Moore L, et al. Child oral health in migrant families: A cross-sectional study of caries in 1-4 year old children from migrant backgrounds residing in Melbourne, Australia. Community Dental Health. 2016;33(2):100-6.
5. Hamid RN, Mudher SH, Ali SM. Caries index, root caries index and gingival index in immigrants at the camp of arbat in sulaimaniya governorate, iraq. International Medical Journal. 2020;27(3):357-9.
6. Hashizume LN, Shinada K, Kawaguchi Y. Factors associated with prevalence of dental caries in Brazilian schoolchildren residing in Japan. J Oral Sci. 2011;53(3):307-12.
7. Lin YC, Chang CS, Ho PS, Lee CH, Chen JH, Huang HL. Immigrant⁻Native Differences in Sugar-Sweetened Beverage and Snack Consumption and Preventive Behaviors Associated with Severe Early Childhood Caries: A Large-Scale Survey in Taiwan. Int J Environ Res Public Health. 2019;16(6).
8. Lin YC, Yen YY, Chang CS, Ting CC, Chen PH, Chen CC, et al. Oral health disparities of children among Southeast Asian immigrant women in arranged transnational marriages in Taiwan. Caries Res. 2014;48(6):575-83.
9. Liu CJ, Zhou W, Feng XS. Dental caries status of students from migrant primary schools in Shanghai Pudong New Area. BMC Oral Health. 2016;16(1).
10. Olerud E, Hagman-Gustavsson ML, Gabre P. Oral health status in older immigrants in a medium-sized Swedish city. Special Care in Dentistry. 2016;36(6):328-34.

11. Sivakumar V, Jain J, Haridas R, Paliayal S, Rodrigues S, Jose M. Oral health status of tibetan and local school children: A comparative study. Journal of Clinical and Diagnostic Research. 2016;10(11):ZC29-ZC33.
12. Stecksén-Blicks C, Hasslöf P, Kieri C, Widman K. Caries and background factors in Swedish 4-year-old children with special reference to immigrant status. Acta Odontol Scand. 2014;72(8):852-8.
13. Wilson FA, Wang Y, Borrell LN, Bae S, Stimpson JP. Disparities in oral health by immigration status in the United States. Journal of the American Dental Association (1939). 2018;149(6):414-21.
14. Dahlan R, Bohlouli B, Salami B, Saltaji H, Amin M. Parental acculturation and oral health of children among immigrants. Journal of public health dentistry. 2021.
15. Diamanti I, Berdouses ED, Kavvadia K, Arapostathis KN, Polychronopoulou A, Oulis CJ. Dental caries prevalence and experience (Icdas ii criteria) of 5-, 12-and 15-year-old children and adolescents with an immigrant background in greece, compared with the host population: A cross-sectional study. International Journal of Environmental Research and Public Health. 2022;19(1).
16. Gómez-Costa D, San-Roman-Montero J, Rojo R, Gil Á, Gómez de Diego R, López-Sánchez AF. Self-reported prevalence of periodontal disease among the Spanish population and immigrants: 2006, 2011/12 and 2017: a population-based study. BMC Oral Health. 2021;21(1).
17. Kabani FA, Stockbridge EL, Berly Varghese B, Loethen AD. Acculturation and the oral health of a nationally representative sample of Hispanic children in the United States: an analysis of 2011-2012 National Survey of Children's Health data. BMC Public Health. 2020;20(1):111.
18. Sanders A, Cardel M, Laniado N, Kaste L, Finlayson T, Perreira K, et al. Diet quality and dental caries in the Hispanic Community Health Study/Study of Latinos. J Public Health Dent. 2020;80(2):140-9.
19. Thorbert-Mros S, Ali N, Ali M, Ayas M, Trullenque-Eriksson A, Dahlén G. A comparative study on periodontitis and periodontitis-associated bacteria in Somali and non-Somali children and adolescents living in Trollhättan, Sweden. European journal of oral sciences. 2021:e12843.
20. Traisuwan W. Oral health status and behaviors of pregnant migrant workers in Bangkok, Thailand: a cross-sectional study. BMC oral health. 2021;21(1):379.
21. Zhang S, Liu J, Lo ECM, Chu CH. Dental caries status of dai preschool children in yunnan province, china. BMC Oral Health. 2013;13(1).
22. Caplin JL, Evans CA, Begole EA. The Relationship between Caries and Malocclusion in Chinese Migrant Workers' Children in Shanghai. Chin J Dent Res. 2015;18(2):103-10.
23. Chen KJ, Liu J, Xu B, Li Y, Li Y, Zhang S, et al. Oral health status of 12-year-old children in Lisu minority ethnic group in China: a cross-sectional study. BMC Oral Health. 2021;21(1):27.
24. Premaraj TS, Vella R, Chung J, Lin Q, Panier H, Underwood K, et al. Ethnic variation of oral microbiota in children. Sci Rep. 2020;10(1):14788.
25. Sgan-Cohen HD, Margvelashvili V, Bilder L, Kalandadze M, Gordon M, Margvelashvili M, et al. Dental caries among children in Georgia by age, gender, residence location and ethnic group. Community Dent Health. 2014;31(3):163-6.
26. Shi C, Faris P, McNeil DA, Patterson S, Potestio ML, Thawer S, et al. Ethnic disparities in children's oral health: findings from a population-based survey of grade 1 and 2 schoolchildren in Alberta, Canada. BMC Oral Health. 2018;18(1):1.
27. Van Der Tas JT, Kragt L, Veerkamp JJS, Jaddoe VWV, Moll HA, Ongkosuwito EM, et al. Ethnic Disparities in Dental Caries among Six-Year-Old Children in the Netherlands. Caries Research. 2016;50(5):489-97.
28. Wu SC, Ma XX, Zhang ZY, Lo ECM, Wang X, Wang B, et al. Ethnic Disparities in Dental Caries among Adolescents in China. J Dent Res. 2021;100(5):496-506.
29. Wulaerhan J, Abudureyimu A, Bao XL, Zhao J. Risk determinants associated with early childhood caries in Uygur children: a preschool-based cross-sectional study. BMC Oral Health. 2014;14:136.
30. Zhang S, Liu J, Lo ECM, Chu CH. Dental and periodontal status of 12-year-old Bulang children in China. BMC Oral Health. 2014;14(1).
31. Zhang S, Xu B, Liu J, Lo ECM, Chu CH. Dental and periodontal status of 12-yearold Dai school children in Yunnan Province, China: A cross-sectional study. BMC Oral Health. 2015;15(1).
32. Hermans MP, Kooistra J, Cannegieter SC, Rosendaal FR, Mook-Kanamori DO, Nemeth B. Healthcare and disease burden among refugees in long-stay refugee camps at Lesbos, Greece. European journal of epidemiology. 2017 Sep;32(9):851-4. (NO DATA ON CARIES PREVELANCE)
33. Williams B, Cassar C, Siggers G, Taylor S. Medical and social issues of child refugees in Europe. Archives of Disease in Childhood. 2016 Sep 1;101(9):839-42.

34. Mattila A, Ghaderi P, Tervonen L, Niskanen L, Pesonen P, Anttonen V, Laitala ML. Self-reported oral health and use of dental services among asylum seekers and immigrants in Finland—a pilot study. The European Journal of Public Health. 2016 Dec 1;26(6):1006-10. Did not divide between participants ref immig all togethrt
35. Vega-López S, Lindberg NM, Eckert GJ, Nicholson EL, Maupome G. Association of added sugar intake and caries-related experiences among individuals of Mexican origin. Community dentistry and oral epidemiology. 2018 Aug;46(4):376-84.
36. Ogawa JT, Kiang J, Watts DJ, Hirway P, Lewis C. Oral health and dental clinic attendance in pediatric refugees. Pediatric dentistry. 2019 Jan 15;41(1):31-4.
37. Alrashdi M, Cervantes Mendez MJ, Farokhi MR. A Randomized Clinical Trial Preventive Outreach Targeting Dental Caries and Oral-Health-Related Quality of Life for Refugee Children. International Journal of Environmental Research and Public Health. 2021 Feb;18(4):1686.
